# Ferrostatin-1 Ameliorates Liver Dysfunction via Reducing Iron in Thioacetamide-induced Acute Liver Injury in Mice

**DOI:** 10.3389/fphar.2022.869794

**Published:** 2022-04-12

**Authors:** Hui Jiang, Xinyu Zhang, Wanping Yang, Meiqi Li, Guohua Wang, Qianqian Luo

**Affiliations:** Department of Physiology and Hypoxic Biomedicine, Institute of Special Environmental Medicine and Co-innovation Center of Neuroregeneration, Nantong University, Nantong, China

**Keywords:** thioacetamide, ferroportin, transferrin receptor 1, ferroptosis inhibitor, acute liver injury, deferoxamine, iron

## Abstract

**Background and Aims:** Hepatic iron overload always leads to oxidative stress, which has been found to be involved in the progression of liver disease. However, whether iron disorder is involved in acute liver disease and the further molecular mechanisms remain unclear.

**Methods:** A mice model of acute liver injury (ALI) was established via intraperitoneal injection of thioacetamide (TAA) (250 mg/kg/day) for 3 consecutive days. Ferrostatin-1 (Fer-1) was administered intraperitoneally (2.5 μM/kg/day) starting 3 days before TAA treatment. Deferoxamine (DFO) was intraperitoneally injected (200 mg/kg/day) with TAA treatment for 3 days. We further observed the effect of Fer-1 on TAA model with high-iron diet feeding. ALI was confirmed using histological examination and liver function activity. Moreover, expressions of iron metabolism and ferroptosis proteins were measured by Western blot analysis.

**Results:** The study revealed that the iron accumulation and ferroptosis contributed to TAA-induced ALI pathogenesis. TAA induced prominent inflammation and vacuolar degeneration in the liver as well as liver dysfunction. In addition, protein expression of the cystine/glutamate antiporter SLC7A11 (xCT) and glutathione peroxidase 4 (GPX4) was significantly decreased in the liver, while transferrin receptor 1 (TfR1), ferroportin (Fpn) and light chain of ferritin (Ft-L) expression levels were increased after TAA exposure. As the same efficiency as DFO, pre-administration of Fer-1 significantly decreased TAA-induced alterations in the plasma ALT, AST and LDH levels compared with the TAA group. Moreover, both Fer-1 and DFO suppressed TfR1, Fpn and Ft-L protein expression and decreased iron accumulation, but did not affect xCT or GPX4 expression in the liver. Both Fer-1and DFO prevented hepatic ferroptosis by reducing the iron content in the liver. Furthermore, Fer-1 also reduced iron and reversed liver dysfunction under iron overload conditions.

**Conclusion:** These findings indicate a role of TAA-induced iron accumulation and ferroptosis in the pathogenesis of ALI model. The effect of Fer-1 was consistent with that of DFO, which prevented hepatic ferroptosis by reducing the iron content in the liver. Thus, Fer-1 might be a useful reagent to reverse liver dysfunction and decreasing the iron content of the liver may be a potential therapeutic strategy for ALI.

## Introduction

The liver is one of the vital organs in the body and plays a vital role in the detoxification of foreign substances, secretion of bile for digestion, metabolic functions of various nutrients, and regulation of iron metabolism. The liver is not only the major iron storage site but also where the iron regulation hormone hepcidin is synthesized (Nicolas et al., 2001; Sikorska et al., 2016). Systemic and cellular iron homeostasis is sustained through several main iron-related proteins, including iron importer, exporter and storage proteins. Iron is taken up into cells via transferrin receptor 1 (TfR1) and sequestered by ferritin light chain (Ft-L) (Hentze et al., 2010) or exported out of cells via ferroportin (Fpn). Iron homeostasis is controlled through regulation of duodenal iron absorption, macrophage iron release and hepatocyte iron storage, which is mainly carried out by the iron-related proteins above (Hentze et al., 2010; Ganz 2011).

Acute liver injury (ALI) often develops rapidly and may involve drug-induced liver failure or cholestasis. Animals that develop ALI show enhanced generation of hepatic ROS and enhanced lipid peroxidation with the formation of lipid peroxides. ALI may be caused by viruses, drugs or toxins. Among the various toxins, a thiono-sulfur-containing compound, thioacetamide (TAA), has been used extensively in the development of animal models of ALI (Chu et al., 2001; Bruck et al., 2007) because it induces lipid peroxidation, oxidative stress, and inflammation, ultimately causing functional hepatocyte death and liver dysfunction (Muller et al., 1991; Grek and Arasi 2016; Thawley 2017).

Ferroptosis, which is a recently identified novel form of regulated cell death, proceeds differently from apoptosis, necrosis, and autophagic cell death (Dixon et al., 2012). It is mediated by the iron-dependent oxidative degeneration of lipids and leads to mitochondrial shrinkage, with increased mitochondrial membrane density and outer mitochondrial membrane rupture (Xie et al., 2016). It has been reported that excess cellular iron is the main driver of the Fenton reaction and the production of reactive oxygen species (ROS) (He et al., 2020). Moreover, ferroptosis was demonstrated to be induced by phospholipid peroxidation associated with free iron-mediated Fenton reactions (Hadian and Stockwell 2020). Thus, we hypothesized that TAA-induced ALI is mediated by excess iron, causing substantial oxidative stress/lipid peroxidation and further inducing ferroptosis in hepatocytes, which finally results in liver injury and dysfunction. In this study, iron and ferroptosis were identified as the cornerstones for detecting the mechanism of ALI. TAA was administered intraperitoneally to induce the ALI model in mice. We found that iron content and mobilization were significantly enhanced, as well as liver dysfunction, while protein expression of the cystine/glutamate antiporter SLC7A11 (xCT) and glutathione peroxidase 4 (GPX4) in the liver was decreased in the TAA-induced ALI model. However, both deferoxamine (DFO) and ferrostatin-1 (Fer-1) suppressed the liver dysfunction induced by TAA by reducing iron accumulation in the liver but did not affect xCT or GPX4 expression. Our data suggest that decreasing the iron content in the liver may be a potential therapeutic strategy for ALI.

## Materials and Methods

### Animals and Reagents

All animals were provided by the animal experimental centre of Nantong University. Mice were maintained in stainless steel cages with a relative humidity of 55–60% at 21 ± 2 C with 12-h rotation periods of light and dark. All animal handling procedures were performed according to approved guidelines. All chemicals and reagents used in this study were purchased from Sigma (St. Louis, MO, United States) unless otherwise stated. The ferroptosis inhibitor ferrostatin-1 (Fer-1) was obtained from Selleck Chemicals (S7243, TX, United States).

### A Mice Model of Acute Liver Injury

Male Institute of Cancer Research mice (ICR, 8 weeks old) were randomly divided into different groups, including control group, TAA group, Fer-1 pre-treatment and TAA group, or TAA with DFO group (*n* = 6-9 mice per group). According to previous studies (França et al., 2019; Han et al., 2019; Liu et al., 2021), TAA was injected (i.p., 250 mg/kg/day) for 3 consecutive days to induce acute liver injury. Fer-1 was administered (i.p., 2.5 μM/kg/day) for 3 days before TAA treatment (Wang et al., 2017), and DFO (200 mg/kg/day, i. p) was injected with TAA for 3 days (Mansour 2000; Wu et al., 2011). TAA, DFO and Fer-1 were all dissolved in normal saline. The control group was injected with equal volumes of normal saline solution, using the same injection schedule. To compare the effects of normal-iron diet (NID) and high-iron diet (HID) on TAA-induced ALI model, we next fed the iron content of diets according to previous protocol (Wang et al., 2017). Briefly, male ICR mice were fed with either a standard AIN-76A diet (50 mg Fe/kg; Research Diets, Inc., New Brunswick, United States) or high-iron AIN-76A diet (8.3 g Fe/kg; Research Diets, Inc.) for 14 days. TAA was administered (i.p., 250 mg/kg/day) for 3 consecutive days after HID intervention. The mice were finally anaesthetized with 1% pentobarbital sodium (40 mg/kg body weight, i. p.) and received myocardial perfusion using phosphate-buffered saline (PBS), after which the liver tissues were collected for measurements.

### Assessment of Liver Functions

Serum levels of alanine aminotransferase (ALT), aspartate aminotransferase (AST), albumin, and glutathione (GSH) were measured following the protocol of an ALT kit (Cat# C009-1-1, Nanjing Jiancheng Bioengineering Institute, Nanjing, China), AST kit (Cat# C010-1-1, Nanjing Jiancheng Bioengineering Institute), albumin kit (Cat# A028-1-1, Nanjing Jiancheng Bioengineering Institute), and GSH assay kit (Cat# A006-1-1, Nanjing Jiancheng Bioengineering Institute), respectively. The optical densities were measured with a Synergy 2 multi-mode microplate reader (Agilent Technologies, Inc.). Each sample was measured three times, and the average absorbance values were calculated for every sample. The concentrations in the samples were determined using the standard curves.

### Hepatic Histopathological Evaluation

After paraformaldehyde fixation, the hepatic tissue was embedded in paraffin and sectioned with at 5-μm thickness. Liver sections were then mounted onto slides and taken for haematoxylin-eosin (H&E) staining. Briefly, slides were deparaffinized in xylene (5 min, twice) and hydrated by passing through decreasing concentration of alcohol baths (100, 90, 80, 70%) and water, followed by haematoxylin staining for 2 min. The sections were then washed in running tap water until sections “blue” for 5 min. The sections were then rinsed with distilled water, rinsed with 0.1% hydrochloric acid in 50% ethanol, rinsed with tap water for 15 min, stained with eosin for 1 min, and rinsed again with tap water. Next, the slides were dehydrated with 95 and 100% ethanol successively followed by xylene (5 min, twice) for clearing. Finally, sections were mounted with neutral balata and covered with a cover-slip.

### Enhanced Perls’ Staining Using Diaminobenzidine

Paraformaldehyde-fixed paraffin-embedded tissues were sectioned into 20 μm sections and stored at room temperature. Slides were blocked for nonspecific binding using a solution block, and endogenous peroxidase activity was quenched. 3,3′-Diaminobenzidine (DAB)-enhanced Perls’ staining was performed to observe iron accumulation in paraffin-embedded liver sections following the manufacturer’s instructions. Briefly, sections of liver tissue were washed with PBS and incubated in freshly prepared Perls’ solution (1% potassium ferricyanide in 0.1-M hydrochloric acid buffer) for 1 h, followed by a 15 min incubation in DAB. Slides were immersed for 1 hour and then stained with DAB. All slides were counterstained with haematoxylin and visualized under a DM4000B microscope (Leica, Germany) at a final magnification of ×200. The data were collected from three fields of view per mice, and analyzed semi-quantitatively with ImageJ software. As described previously (Moos and Møllgård 1993), the quantitative analysis of Perls’ staining was used to reflect iron deposition levels. Integrated Density of different treatment groups was finally normalized to control group. The analysis was done by an investigator masked to experimental group.

### Western Blotting

Proteins were collected and homogenized in RIPA lysis buffer (Beyotime, PRC) and sonicated with a sonifier. The BCA (Pierce, Rockford) detection method was employed to detect protein content. A sample containing 30 μg protein was loaded and run in each well of SDS–PAGE gels. The membranes were incubated with primary antibodies (1:1,000) against TfR1 (Cat. 13–6800, Thermo Fisher Scientific, MA, United States), Fpn1 (Cat. NBP1-21502, Novus, Centennial, CO, United States), ferritin-L (Cat. 10727-1-AP, Proteintech, Chicago, United States), xCT (Cat. 26864-1-AP, Proteintech) and GPx4 (Cat. ab125066, Abcam, Cambridge, United Kingdom) at 4°C overnight. Blots were then incubated with goat anti-rabbit or anti-mouse IRDye 800 CW secondary antibody at a 1:10,000 dilution (Li-Cor, Lincoln Co., Ltd., United States) at room temperature for 1 h. GAPDH (MAB374, Merck, State of New Jersey, United States) monoclonal antibody (1:10,000) was used as a loading control. The band densities of the specific blots were scanned via Odyssey CLx infrared imaging system (LI-COR Biosciences) and analyzed with ImageJ software. Results were shown as the optical density ratio normalized to GAPDH.

### Tissues Iron Measurements

Iron in the liver was detected using the tissue iron measurement method as follows. Liver tissue (0.1 g) was obtained, and then, 1 ml of tissue digestive liquid (3 M hydrochloric acid and 0.61 M trichloroacetic acid) was added to the liver for digestion at 65 C for 60 h to ensure complete digestion of the liver tissue. After digestion, the volume of the tissue digestion liquid was fixed to 1.5 ml, and the digestion mixture was centrifuged at 10,000 g for 10 min. The supernatant was removed and collected for detection. Iron developing color working solution was freshly prepared for each experiment, and consisted of 100 mg disodium-4,7-diphenyl-1,10-phenanthroline disulfonate (Cat. 146617, sigma, State of New Jersey, United States), 60 ml ddH2O, 1.429 ml 70% thioglycollic acid (S8750, sigma, State of New Jersey, United States), supplement with ddH2O to 100 ml. A 96-well plate was further used for detection, and 200 ml iron developing colour working solution was added to each well. Then, 10 ml ddH2O, 10 ml tissue digestion solution or 10 ml iron standard solution (500 μg/dl) was added to different wells. Finally, 10 ml of sample was added to the sample well, fully mixed, and incubated at room temperature for 10 min. Absorbance was measured at 535 nm. The method of iron quantification was according to the formula, iron content (Ug/g Tissue wet weight) = OD/tissue weight × (1.5–0.25 × tissue weight) × (1/iron standard 500 OD × 4.77).

### Statistical Analysis

Statistical analysis was performed with GraphPad Prism 8.0 software. All the data in this study are shown as the mean ± SEM. Data of two groups were analyzed for statistical significance with Student’s t-test (non-directional). Variations between the means in multiple groups were analysed via one-way analysis of variance, and then, Tukey’s post-hoc test was performed for multiple comparisons. A probability value of *p* < 0.05 was viewed as statistically significant.

## Results

TAA induced liver dysfunction, suppressed anti-ferroptosis-related protein expression and enhanced iron-related protein expression in the liver.

**FIGURE 1:**
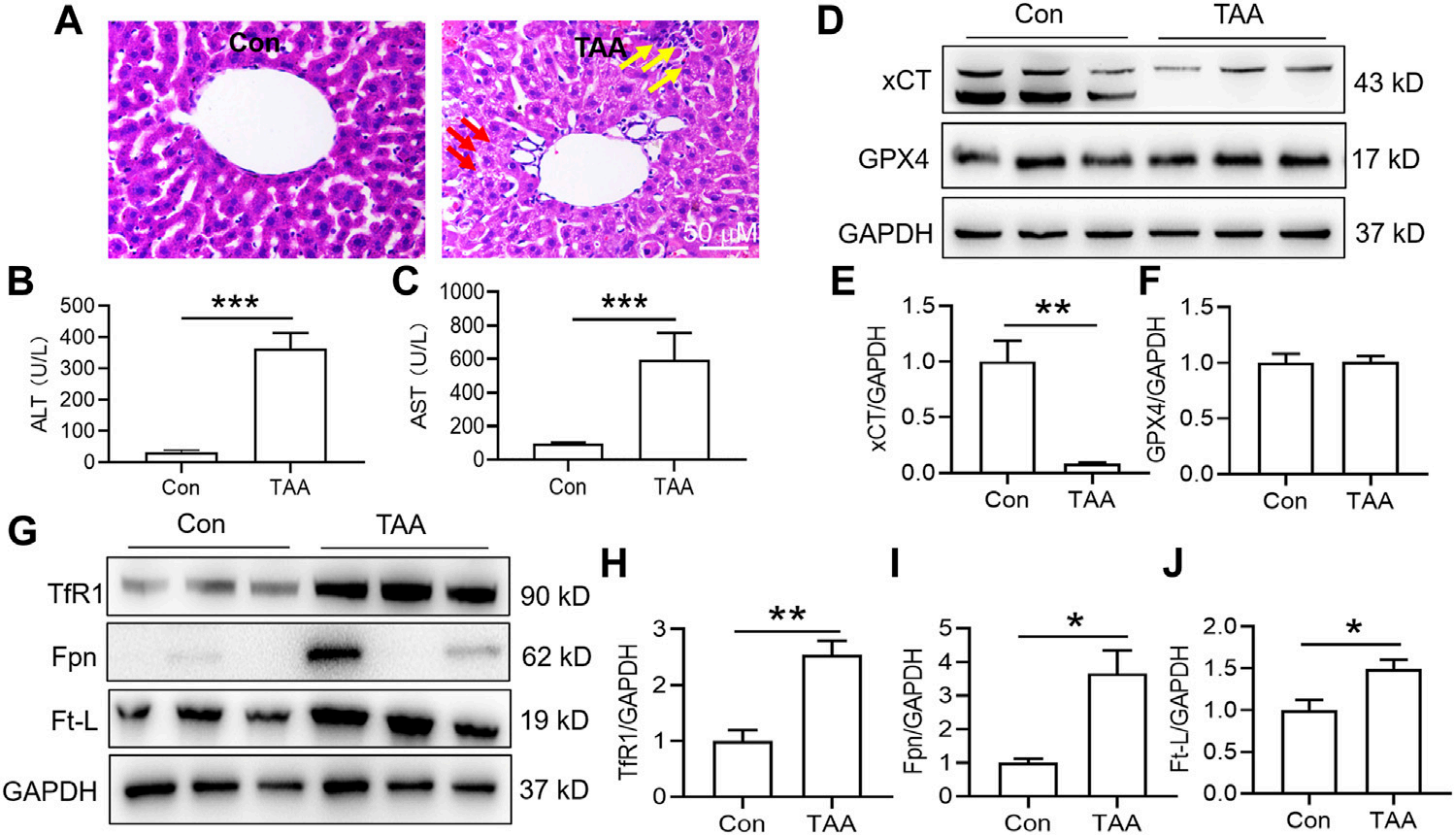
TAA induced liver dysfunction, suppressed anti-ferroptosis-related protein expression and enhanced iron-related protein expression in the liver. **(A)** Liver injury was assessed by H&E staining and histological examination in mice with or without TAA administration for 3 days (bar = 50 μm). TAA induced prominent inflammation (yellow arrow) and vacuolar degeneration (red arrow) in the liver within 3 days compared with the control group. **(B)** Plasma ALT and **(C)** AST levels in control and TAA-injected mice. **(D)** Western blotting analysis of xCT and GPX4 expression in the livers of mice with or without TAA treatment. **(E–F)** Relative protein expression levels of xCT and GPX4. **(G)** Western blotting analysis of TfR1, Fpn and Ft-L expression in the livers of mice with or without TAA treatment. **(H–J)** Relative protein expression levels of TfR1, Fpn and Ft-L. All data are presented as the mean ± SEM (n = 6-9 mice per group); *p < 0.05, **p < 0.01 and ***p < 0.001 versus the indicated group.

Injection of TAA induced prominent inflammation and vacuolar degeneration in the liver within 3 days compared with the control group (Figure 1A). TAA induced obvious liver dysfunction, which was reflected by increased plasma ALT and AST levels in the TAA model (Figures 1B,C, *p* < 0.001). In addition, TAA significantly reduced anti-ferroptosis-related protein (xCT) expression but not GPX4 expression compared to the control (Figures 1D–F). In addition, TAA induced a significant increase in iron-related proteins, including TfR1, Fpn and Ft-L expression, compared to the control (Figures 1G–J, *p* < 0.05 or *p* < 0.01).

Both Fer-1 and DFO suppressed TAA-induced liver dysfunction but did not affect anti-ferroptosis-related protein expression in the liver.

**FIGURE 2:**
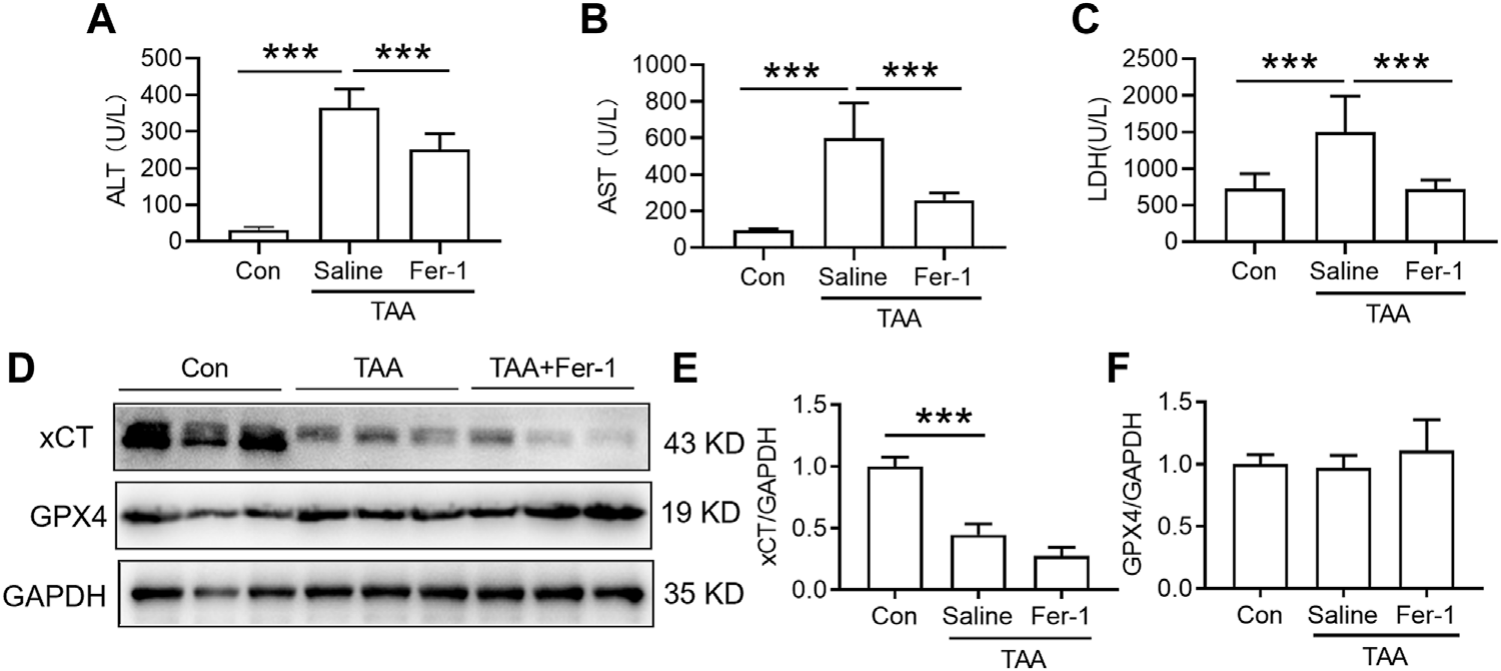
Effects of Fer-1 on liver function and anti-ferroptosis-related protein expression in TAA-induced ALI mice. **(A)** Plasma ALT, **(B)** AST and **(C)** LDH levels in the control and TAA model mice with or without Fer-1 pre-treatment. **(D)** Western blotting analysis of xCT and GPX4 expression in the liver of control and ALI model mice with or without Fer-1 pre-treatment. **(E–F)** Relative protein expression levels of xCT and GPX4 expression in **(D)**. All data are presented as the mean ± SEM (*n* = 6-9 mice per group); ***p < 0.001 versus the indicated group.

In comparison with the control, injection of TAA induced a profound increase in plasma levels of ALT and AST concomitant with a marked increase in the plasma LDH level. Pre-administration of Fer-1 for 3 days significantly decreased TAA-induced alterations in the plasma ALT, AST and LDH levels compared with the TAA group (Figures 2A–C, *p* < 0.001). TAA significantly reduced xCT but not GPX4 expression in the livers of mice. However, pre-administration of Fer-1 did not affect xCT or GPX4 expression in the liver compared to TAA-treated mice (Figures 2D–F).

**FIGURE 3:**
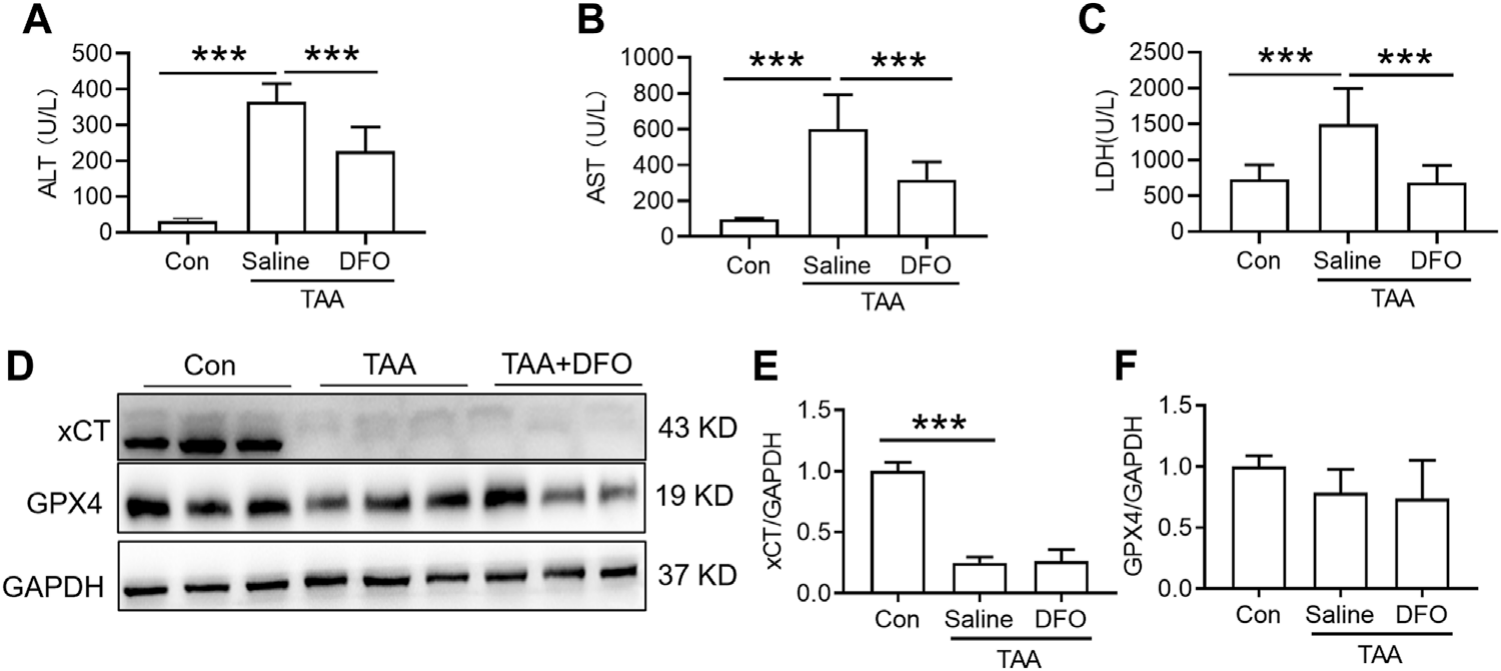
Effects of DFO on liver function and anti-ferroptosis-related protein expression in TAA-induced ALI mice. **(A)** Plasma ALT, **(B)** AST and **(C)** LDH levels in the control and TAA model mice with or without DFO treatment. **(D)** Western blotting analysis of xCT and GPX4 expression in the livers of the control and ALI model mice with or without DFO treatment. **(E–F)** Relative protein expression of xCT and GPX4 in **(D)**. All data are presented as the mean ± SEM (*n* = 6-9 mice per group); ***p < 0.001 versus the indicated group.

Administration of DFO for 3 days with TAA treatment significantly attenuated the TAA-induced increase in plasma ALT, AST and LDH (Figures 3A–C, *p* < 0.001). In addition, administration of DFO did not alter xCT or GPX4 expression induced by TAA in the liver compared to TAA-treated mice (Figures 3D–F).

### Both Fer-1 and Deferoxamine Reduced Thioacetamide-Induced Iron Accumulation in Liver

**FIGURE 4:**
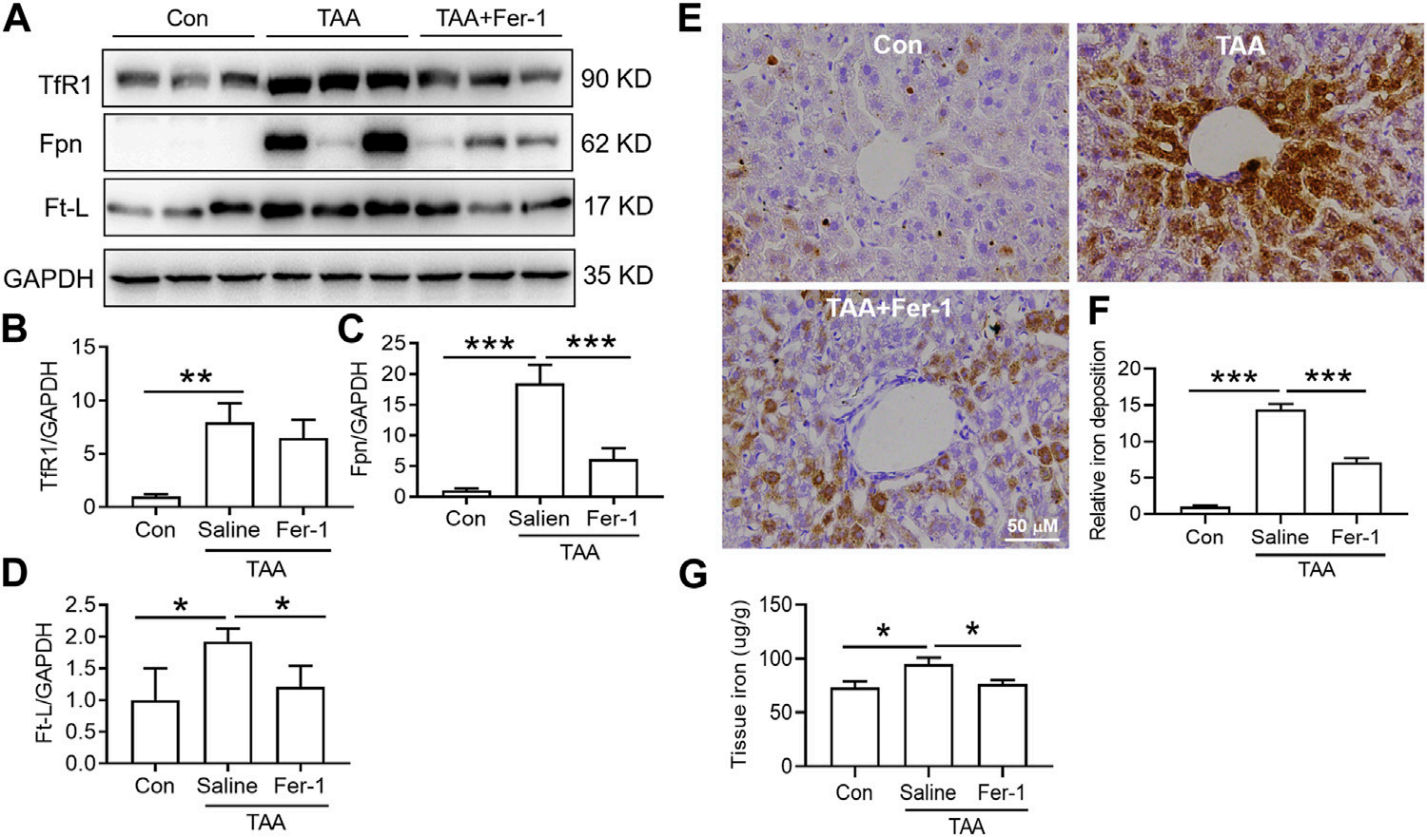
Effects of Fer-1 on iron-related protein expression and iron content in TAA-induced ALI mice. **(A)** Western blotting analysis of TfR1, Fpn and Ft-L expression in the livers of mice after different treatments. **(B–D)** Relative protein expression of TfR1, Fpn and Ft-L in **(A) (E)** Fer-1 suppressed the increase in positive centrolobular iron deposition induced by TAA. **(F)** Semiquantitative iron levels in the mouse livers described in **(E) (G)** Iron content detection in the livers of mice with different treatments. All data are presented as the mean ± SEM (*n* = 6-9 mice per group); *p < 0.05, **p < 0.01, and ***p < 0.001 versus the indicated group.

TAA induced a significant increase in iron uptake, iron export and iron storage in the liver, which were reflected by increased TfR1, Fpn and Ft-L expression, respectively (Figures 4A–D). In addition, iron staining of the liver showed that TAA induced significant iron accumulation (Figures 4E,F, *p* < 0.001), especially positive centrolobular deposition of iron and liver nonheme iron content (Figure 4G, *p* < 0.05). However, Fer-1 pre-treatment reduced the TAA-induced increase in Fpn and Ft-L but did not affect TfR1 expression. In addition, compared to TAA alone, Fer-1 pre-treatment decreased the TAA-induced iron levels and increased iron accumulation in the liver (Figure 4).

**FIGURE 5:**
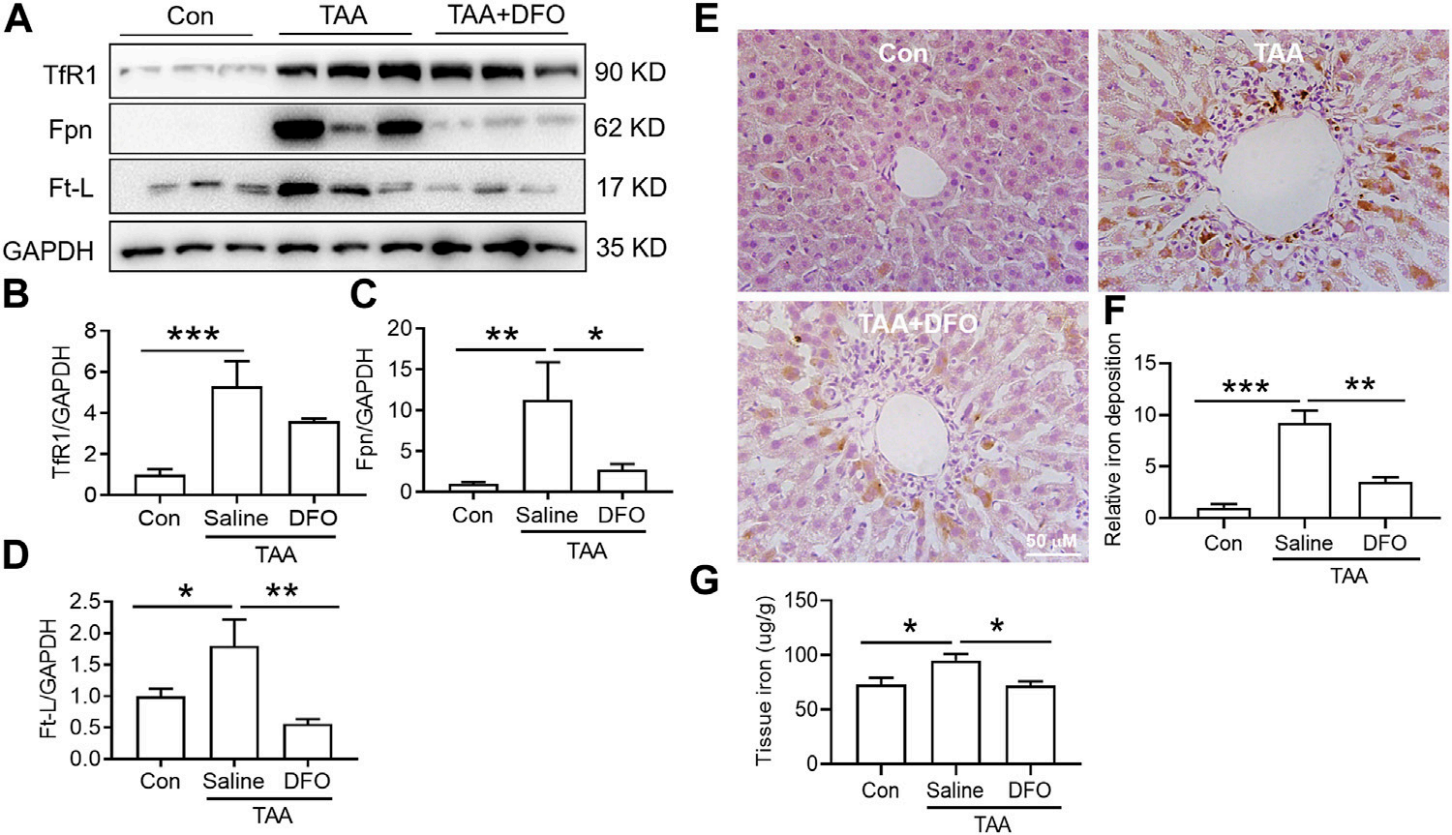
Effects of DFO on iron-related protein expression and iron content in TAA-induced ALI mice. **(A)** Western blotting analysis of TfR1, Fpn and Ft-L expression in the livers of mice with different treatments. **(B–D)** Relative protein exprssion of TfR1, Fpn and Ft-L in **(A) (E)** DFO suppressed the increase in positive centrolobular iron deposition induced by TAA. **(F)** Semiquantitative iron levels of the mice liver in **(E) (G)** Iron content detection in the livers of mice with different treatments. All data are presented as the mean ± SEM (*n* = 6-9 mice per group); *p < 0.05, **p < 0.01, and ***p < 0.001 versus the indicated group.

TAA similarly upregulated TfR1, Fpn and Ft-L expression in the liver (Figures 5A–D, *p* < 0.01 or *p* < 0.05), and also induced significant iron accumulation (Figures 5E,F, *p* < 0.001) and increased liver iron content (Figure 5G, *p* < 0.05). However, DFO pre-treatment before TAA administration significantly decreased Fpn and Ft-L but did not affect TfR1 expression (Figures 5A–D). Additionally, DFO decreased the TAA-induced increase in the iron level and iron accumulation in the liver (Figures 5E–G).

Fer-1 reversed TAA-induced liver dysfunction by reducing iron but not activating anti-ferroptosis-related protein expression in the liver under iron overload conditions.

**FIGURE 6:**
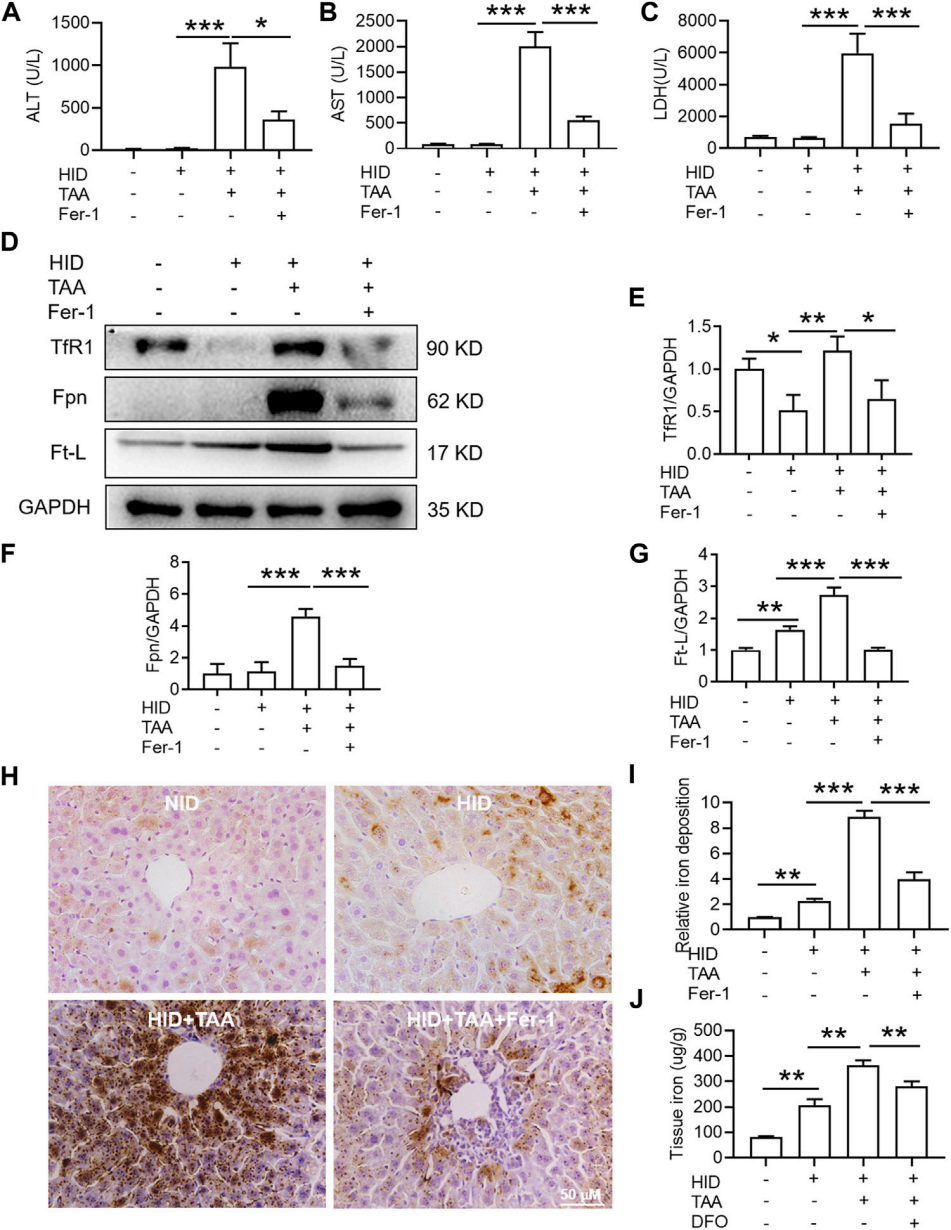
Effects of Fer-1 on liver function, hepatic iron content and anti-ferroptosis-related protein expression in the liver under iron overload conditions. **(A)** Plasma ALT **(B)** AST and **(C)** LDH levels in the control and TAA model mice with or without Fer-1 pre-treatment under high iron diet (HID) conditions. **(D)** Western blotting analysis of TfR1, Fpn and Ft-L expression in the livers of mice with different treatments under HID conditions. **(E–G)** Relative protein expression of TfR1, Fpn and Ft-L in **(D) (H)** Fer-1 inhibited the increase in positive centrolobular iron deposition induced by TAA under HID conditions in the liver. **(I)** Semiquantitative analysis of iron deposition levels in **(H) (J)** Iron content detection in the livers of mice with different treatments. All data are presented as the mean ± SEM (*n* = 6-9 mice per group); *p < 0.05, **p < 0.01, and ***p < 0.001 versus the indicated group.

Compared with NID, HID did not increase the levels of hepatic damage biomarkers (ALT, AST and LDH), and this diet was not inducing the same acute liver damage as TAA (Figures 6A–C). In addition, HID downregulated the protein expression of TfR1 (Figures 6D,E, *p* < 0.05), but did not influence the expression of Fpn compared to NID (Figures 6D,F). Unlike TfR1 level, Ft-L expression was significantly enhanced by HID in the liver (Figure 6G, *p* < 0.01). HID-fed mice had developed a significant iron increase than NID mice (Figures 6H–J, *p* < 0.01).

Consistent with the effects of TAA under NID conditions, TAA induced profound liver dysfunction, which was shown by an increase in plasma ALT, AST and LDH levels under iron overload conditions (Figures 6A–C). Pre-administration of Fer-1 for 3 days before TAA injection significantly attenuated the TAA-induced increase in plasma ALT, AST and LDH compared with the TAA treatment group under high iron conditions (Figures 6A–C). Similar to TAA treatment under NID conditions, TAA induced a significant enhancement in TfR1, Fpn and Ft-L expression in the liver (Figures 6D–G). Moreover, compared to the HID group, iron staining results suggested that TAA induced significant iron accumulation under high iron conditions (Figures 6H,I) and increased liver iron detection (Figure 6J). However, pre-treatment of Fer-1 reversed the increases in the expression levels of the above three proteins and iron accumulation in the liver under HID conditions (Figures 6D–J).

## Discussion

TAA is a potent toxicant that causes oxidative stress and further induces liver damage, which is highly similar to human acute liver damage (Zargar et al., 2019). The TAA-induced liver injury model has been widely studied for its biochemical and histological effects in animals and is usually used to establish liver injury models (Rahman and Hodgson 2003). However, administration of TAA to mice causes either chronic/acute liver failure or cirrhosis depending on the dose and duration of TAA exposure (Shapiro et al., 2006). In previous studies, high-dosage TAA is known to induce lipid peroxidation and oxidative stress, and the pathological effects are mainly limited to ALI rather than causing direct damage to other organs (Muller et al., 1991; Mladenovic et al., 2012). Thus, according to previous studies (França et al., 2019; Han et al., 2019; Liu et al., 2021), we administered 250 mg/kg/day TAA to mice via intraperitoneal injection for 3 days to establish the ALI model in our present study.

Although TAA-induced ALI was primarily provoked by oxidative stress (Zhan et al., 2019), whether it is partially an iron-dependent process is still uncertain. To investigate whether iron disorder is involved in liver dysfunction, we evaluated liver function and liver iron content in TAA-induced ALI mice. Because the liver is the major extraerythrocyte storage organ for iron and most of the iron within the liver is stored in Ft-L, which is often used to indicate iron levels (Ishizaka et al., 2005; Galaris and Pantopoulos 2008). TAA not only significantly induced iron mobilization, which was reflected as increased transferrin receptor 1 (TfR1), but also increased iron storage, which was reflected as increased ferritin-L (Ft-L) expression in the liver. These results were consistent with those of other studies suggesting that TAA induces iron accumulation in the liver of ALI mice (Ackerman et al., 2015). However, we also noticed that TAA induced the increase of ferroportin (Fpn) expression in the liver, which usually facilitate iron efflux to lower tissue iron. Actually, iron is tightly controlled at cell and systemic levels to prevent both deficiency and overload under physiological conditions. Iron regulatory proteins post-transcriptionally control genes encoding proteins that modulate iron transport, use and storage and are themselves regulated by iron (Gao et al., 2019). Thus, the elevated Fpn may represent an endogenous effort to maintain iron homoeostasis (Anderson and Frazer 2017). It is also understandable that both Fer-1 and DFO decreased iron content, while reducing Fpn in the liver after TAA exposure. Intracellular iron can be stored in the protein shell of ferritin (Ft) as a crystalline core of ferric (Fe^3+^) ions, and the iron must first be released from the core to catalyze oxidative reactions (Skaper 2019). Thus, Ft is able to restrict the availability of iron to participate in redox active iron species in the cytosol. However, more ferritin subunits are synthesized using translational and transcriptional mechanisms in response to increased cell iron (Badu-Boateng and Naftalin 2019). Increased intracellular ferritin may occur in response to oxidative stress provoked by accumulated iron, which could be result of various stressors such as UV light, increased temperature, and inflammation (Vile and Tyrrell 1993; Tisma et al., 2009). Consistent with previous findings, we found that TAA significantly induced iron increase in the liver and also promoted Ft-L expression.

Although ferroptosis was reported to be involved in the pathogenesis of many injury models (Skouta et al., 2014; Liu et al., 2020), it is still unclear whether ferroptosis is involved in the TAA-induced ALI model. In addition, iron accumulation is considered to be the main cause of ferroptosis initiation (Li et al., 2020). This prompted us to wonder whether TAA induces iron accumulation and ultimately initiates ferroptosis in the liver. The synthetic antioxidant Fer-1 was reported to act as a ferroptosis inhibitor via a reductive mechanism to prevent damage to membrane lipids, thereby inhibiting ferroptosis (Dixon et al., 2012). The iron chelator DFO is widely used to reduce iron in tissue after injection or oral administration (Buss et al., 2003). In the present study, we introduced Fer-1 and DFO to investigate whether ferroptosis occurred because these agents can decrease iron content to achieve an anti-ferroptotic effect in the TAA-induced ALI model. We found that both Fer-1 and DFO rescued liver dysfunction and inhibited iron content and accumulation in the liver. However, it was previously reported that Fer-1 can attenuate oxidative, iron-dependent cancer cell death by blocking cystine import and glutathione production (Skouta et al., 2014). Our data demonstrated that Fer-1 and DFO did not play anti-ferroptotic roles by affecting cystine import or glutathione production in a TAA-induced ALI model. This result may suggest that Fer-1 plays an anti-ferroptotic role similar to DFO in the liver in the ALI model and that the mechanism involves chelation of hepatic iron. Moreover, a recent study demonstrated that Fer-1, in the presence of ferrous iron, produces the most relevant anti-ferroptotic effect by forming a complex with iron (Miotto et al., 2020).

Ferroptosis is a multi-step regulated cell death that is characterized by excessive iron accumulation and lipid peroxidation (Yu et al., 2020). Lipid peroxide accumulation is mainly through the xCT and GPX4-dependent mechanisms (Kim et al., 2021). The process of ferroptosis is also dependent on intracellular iron because the accumulation of iron acts as a catalyst for converting peroxides into free radicals (Kim et al., 2021). Oxidative degradation of lipids occurs when there is depletion of the antioxidant glutathione and a loss of activity of the lipid repair enzyme GPX4. Lipid peroxidation then leads to cell membrane denaturation (Cao and Dixon 2016). The results were also consistent with other studies, which confirmed that ferroptosis can be induced by a loss of activity of xCT and suppression of GPX4, followed by accumulation of lipid reactive oxygen species (ROS) (Ma et al., 2008; Yang and Stockwell 2016; Latunde-Dada 2017). However, both anti-ferroptotic proteins xCT and GPX4 were not modulated by Fer-1 investigated. It was concluded that the outcome of TAA-induced acute liver injury may depend on the level of hepatic iron concentration and iron overload may exacerbate the injury. However, it’s not entirely clear that whether Fer-1 or DFO block iron mobilisation into the liver instead. Thus, more study is necessary to investigate their effects on the expression of molecules that play key roles in iron transport, use and storage.

**FIGURE 7:**
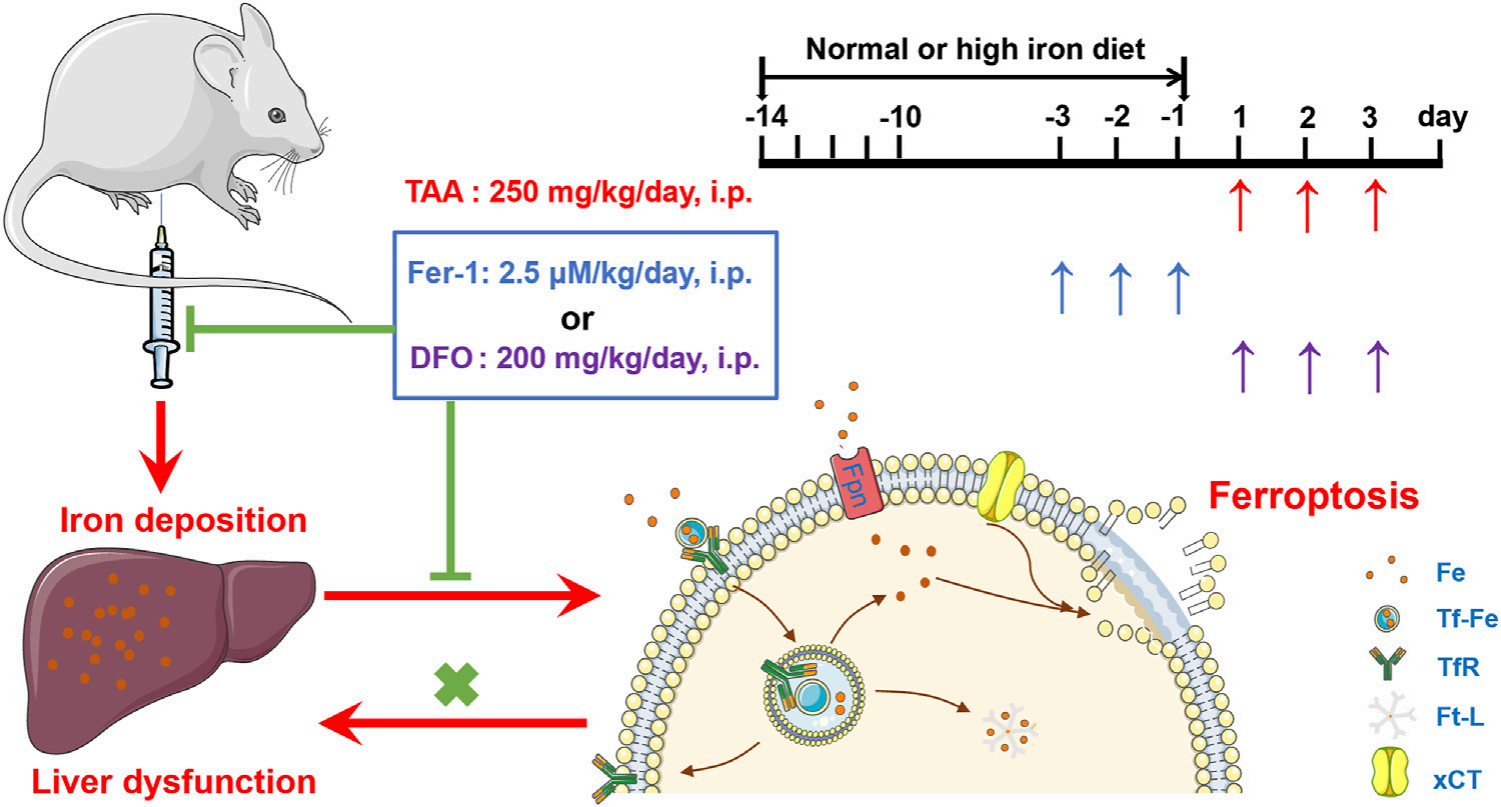
Proposed mechanisms of the ferroptosis inhibitor Fer-1 against the TAA-induced ALI mice model.

In addition, the study revealed HID-fed mice had developed a significant iron increase. In addition, HID did not increase the protein expression of TfR1 and Fpn in the same magnitude of TAA. Indeed, HID even downregulated TfR1 compared to NID. Similar with previous research (Weiss et al., 2018), the decreased TfR1 after HID may also represent endogenous feedback to maintain the iron balance. Unlike TfR1 level, Ft-L expression was significantly enhanced by HDI in the liver, which directly indicated increased iron levels. We further found that HID did not increase the levels of hepatic damage biomarkers (ALT, AST and LDH), and this diet was not inducing the same acute liver damage as TAA. However, previous study has revealed that an increase in hepatic iron concentration might exacerbate TAA-induced live injury (Ackerman et al., 2015). We confirmed that TAA aggravated the damage to the liver with iron overload. We further revealed that Fer-1 still had an anti-ferroptotic effect on TAA-induced liver ferroptosis by reducing the iron content in high iron-fed ALI mice. All these results provide new evidence of the anti-ferroptotic effect of Fer-1 in the TAA-induced ALI model, the mechanism of which is mediated by the ability of Fer-1 to reduce iron content and iron accumulation in the liver (Figure 7).

Taken together, these findings indicate a role of TAA-induced iron deposition and ferroptosis in the pathogenesis of ALI model. Ferroptosis inhibitor Fer-1 plays a role in rescuing liver injury and dysfunction by reducing liver iron levels and inhibiting TAA-induced ferroptosis in the liver. Similar to DFO, Fer-1 might be a useful reagent to reverse liver dysfunction under iron overload conditions.

## Data Availability

The raw data supporting the conclusions of this article will be made available by the authors, without undue reservation.

## Glossary

ALI: acute liver injury
ALT: alanine aminotransferase
AST: aspartate aminotransferase
DFO: deferoxamine
Fer-1: ferrostatin-1
Fpn: ferroportin
Ft-L: ferritin-L
GPX4: glutathione peroxidase 4
GSH: aseglutathione
TAA: thioacetamide
TfR1: transferrin receptor 1
xCT: cystine/glutamate antiporter SLC7A11

## References

[B1] Ackerman Z. Pappo O. Link G. Glazer M. Grozovski M. (2015). Liver Toxicity of Thioacetamide Is Increased by Hepatocellular Iron Overload. Biol. Trace Elem. Res. 163 (1-2), 169–176. 10.1007/s12011-014-0110-9 25161090

[B2] Anderson G. J. Frazer D. M. (2017). Current Understanding of Iron Homeostasis. Am. J. Clin. Nutr. 106 (Suppl. 6), 1559s–1566s. 10.3945/ajcn.117.155804 29070551 PMC5701707

[B3] Badu-Boateng C. Naftalin R. J. (2019). Ascorbate and Ferritin Interactions: Consequences for Iron Release *In Vitro* and *In Vivo* and Implications for Inflammation. Free Radic. Biol. Med. 133, 75–87. 10.1016/j.freeradbiomed.2018.09.041 30268889

[B4] Bruck R. Ashkenazi M. Weiss S. Goldiner I. Shapiro H. Aeed H. (2007). Prevention of Liver Cirrhosis in Rats by Curcumin. Liver Int. 27 (3), 373–383. 10.1111/j.1478-3231.2007.01453.x 17355460

[B5] Buss J. L. Torti F. M. Torti S. V. (2003). The Role of Iron Chelation in Cancer Therapy. Curr. Med. Chem. 10 (12), 1021–1034. 10.2174/0929867033457638 12678674

[B6] Cao J. Y. Dixon S. J. (2016). Mechanisms of Ferroptosis. Cell Mol Life Sci 73 (11-12), 2195–2209. 10.1007/s00018-016-2194-1 27048822 PMC4887533

[B7] Chu C. J. Wang S. S. Lee F. Y. Chang F. Y. Lin H. C. Hou M. C. (2001). Detrimental Effects of Nitric Oxide Inhibition on Hepatic Encephalopathy in Rats with Thioacetamide-Induced Fulminant Hepatic Failure. Eur. J. Clin. Invest. 31 (2), 156–163. 10.1046/j.1365-2362.2001.00775.x 11168455

[B8] Dixon S. J. Lemberg K. M. Lamprecht M. R. Skouta R. Zaitsev E. M. Gleason C. E. (2012). Ferroptosis: an Iron-dependent Form of Nonapoptotic Cell Death. Cell 149 (5), 1060–1072. 10.1016/j.cell.2012.03.042 22632970 PMC3367386

[B9] França M. E. R. Ramos R. K. L. G. Oliveira W. H. Duarte-Silva E. Araújo S. M. R. Lós D. B. (2019). Tadalafil Restores Long-Term Memory and Synaptic Plasticity in Mice with Hepatic Encephalopathy. Toxicol. Appl. Pharmacol. 379, 114673. 10.1016/j.taap.2019.114673 31323263

[B10] Galaris D. Pantopoulos K. (2008). Oxidative Stress and Iron Homeostasis: Mechanistic and Health Aspects. Crit. Rev. Clin. Lab. Sci. 45 (1), 1–23. 10.1080/10408360701713104 18293179

[B11] Ganz T. (2011). Hepcidin and Iron Regulation, 10 Years Later. Blood 117 (17), 4425–4433. 10.1182/blood-2011-01-258467 21346250 PMC3099567

[B12] Gao G. Li J. Zhang Y. Chang Y. Z. (2019). Cellular Iron Metabolism and Regulation. Adv. Exp. Med. Biol. 1173, 21–32. 10.1007/978-981-13-9589-5_2 31456203

[B13] Grek A. Arasi L. (2016). Acute Liver Failure. AACN Adv. Crit. Care 27 (4), 420–429. 10.4037/aacnacc2016324 27959298

[B14] Hadian K. Stockwell B. R. (2020). SnapShot: Ferroptosis. Cell 181 (5), 1188–e1. 10.1016/j.cell.2020.04.039 32470402 PMC8157339

[B15] Han J. H. Kim O. H. Lee S. C. Kim K. H. Park J. H. Lee J. I. (2019). A Novel Hepatic Anti-fibrotic Strategy Utilizing the Secretome Released from Etanercept-Synthesizing Adipose-Derived Stem Cells. Int. J. Mol. Sci. 20 (24), 6302. 10.3390/ijms20246302 PMC6940971 31847135

[B16] He Y. J. Liu X. Y. Xing L. Wan X. Chang X. Jiang H. L. (2020). Fenton Reaction-independent Ferroptosis Therapy via Glutathione and Iron Redox Couple Sequentially Triggered Lipid Peroxide Generator. Biomaterials 241, 119911. 10.1016/j.biomaterials.2020.119911 32143060

[B17] Hentze M. W. Muckenthaler M. U. Galy B. Camaschella C. (2010). Two to Tango: Regulation of Mammalian Iron Metabolism. Cell 142 (1), 24–38. 10.1016/j.cell.2010.06.028 20603012

[B18] Ishizaka N. Saito K. Noiri E. Sata M. Ikeda H. Ohno A. (2005). Administration of ANG II Induces Iron Deposition and Upregulation of TGF-Beta1 mRNA in the Rat Liver. Am. J. Physiol. Regul. Integr. Comp. Physiol. 288 (4), R1063–R1070. 10.1152/ajpregu.00281.2004 15604307

[B19] Kim S. Kang S. W. Joo J. Han S. H. Shin H. Nam B. Y. (2021). Characterization of Ferroptosis in Kidney Tubular Cell Death under Diabetic Conditions. Cell Death Dis 12 (2), 160. 10.1038/s41419-021-03452-x 33558472 PMC7870666

[B20] Latunde-Dada G. O. (2017). Ferroptosis: Role of Lipid Peroxidation, Iron and Ferritinophagy. Biochim. Biophys. Acta Gen. Subj 1861 (8), 1893–1900. 10.1016/j.bbagen.2017.05.019 28552631

[B21] Li J. Cao F. Yin H. L. Huang Z. J. Lin Z. T. Mao N. (2020). Ferroptosis: Past, Present and Future. Cel Death Dis 11 (2), 88. 10.1038/s41419-020-2298-2 PMC6997353 32015325

[B22] Liu P. Feng Y. Li H. Chen X. Wang G. Xu S. (2020). Ferrostatin-1 Alleviates Lipopolysaccharide-Induced Acute Lung Injury via Inhibiting Ferroptosis. Cell Mol Biol Lett 25, 10. 10.1186/s11658-020-00205-0 32161620 PMC7045739

[B23] Liu R. Cui J. Sun Y. Xu W. Wang Z. Wu M. (2021). Autophagy Deficiency Promotes M1 Macrophage Polarization to Exacerbate Acute Liver Injury via ATG5 Repression during Aging. Cell Death Discov 7 (1), 397. 10.1038/s41420-021-00797-2 34930917 PMC8688512

[B24] Ma J. Chen J. Y. Idowu M. Nyokong T. (2008). Generation of Singlet Oxygen via the Composites of Water-Soluble Thiol-Capped CdTe Quantum Dots-Sulfonated Aluminum Phthalocyanines. J. Phys. Chem. B 112 (15), 4465–4469. 10.1021/jp711537j 18363400

[B25] Mansour M. A. (2000). Protective Effects of Thymoquinone and Desferrioxamine against Hepatotoxicity of Carbon Tetrachloride in Mice. Life Sci. 66 (26), 2583–2591. 10.1016/s0024-3205(00)00592-0 10883736

[B26] Miotto G. Rossetto M. Di Paolo M. L. Orian L. Venerando R. Roveri A. (2020). Insight into the Mechanism of Ferroptosis Inhibition by Ferrostatin-1. Redox Biol. 28, 101328. 10.1016/j.redox.2019.101328 31574461 PMC6812032

[B27] Mladenovic D. Krstic D. Colovic M. Radosavljevic T. Rasic-Markovic A. Hrncic D. (2012). Different Sensitivity of Various Brain Structures to Thioacetamide-Induced Lipid Peroxidation. Mc 8 (1), 52–58. 10.2174/157340612799278603 22420551

[B28] Moos T. Møllgård K. (1993). A Sensitive post-DAB Enhancement Technique for Demonstration of Iron in the central Nervous System. Histochemistry 99 (6), 471–475. 10.1007/bf00274100 7691783

[B29] Müller D. Sommer M. Kretzschmar M. Zimmermann T. Buko V. U. Lukivskaya O. (1991). Lipid Peroxidation in Thioacetamide-Induced Macronodular Rat Liver Cirrhosis. Arch. Toxicol. 65 (3), 199–203. 10.1007/BF02307309 2053847

[B30] Nicolas G. Bennoun M. Devaux I. Beaumont C. Grandchamp B. Kahn A. (2001). Lack of Hepcidin Gene Expression and Severe Tissue Iron Overload in Upstream Stimulatory Factor 2 (USF2) Knockout Mice. Proc. Natl. Acad. Sci. U S A. 98 (15), 8780–8785. 10.1073/pnas.151179498 11447267 PMC37512

[B31] Rahman T. M. Hodgson H. J. (2003). The Effects of Early and Late Administration of Inhibitors of Inducible Nitric Oxide Synthase in a Thioacetamide-Induced Model of Acute Hepatic Failure in the Rat. J. Hepatol. 38 (5), 583–590. 10.1016/s0168-8278(03)00050-3 12713868

[B32] Shapiro H. Ashkenazi M. Weizman N. Shahmurov M. Aeed H. Bruck R. (2006). Curcumin Ameliorates Acute Thioacetamide-Induced Hepatotoxicity. J. Gastroenterol. Hepatol. 21 (2), 358–366. 10.1111/j.1440-1746.2005.03984.x 16509859

[B33] Sikorska K. Bernat A. Wroblewska A. (2016). Molecular Pathogenesis and Clinical Consequences of Iron Overload in Liver Cirrhosis. Hepatobiliary Pancreat. Dis. Int. 15 (5), 461–479. 10.1016/s1499-3872(16)60135-2 27733315

[B34] Skaper S. D. (2019). Oligodendrocyte Precursor Cells as a Therapeutic Target for Demyelinating Diseases. Prog. Brain Res. 245, 119–144. 10.1016/bs.pbr.2019.03.013 30961866

[B35] Skouta R. Dixon S. J. Wang J. Dunn D. E. Orman M. Shimada K. (2014). Ferrostatins Inhibit Oxidative Lipid Damage and Cell Death in Diverse Disease Models. J. Am. Chem. Soc. 136 (12), 4551–4556. 10.1021/ja411006a 24592866 PMC3985476

[B36] Thawley V. (2017). Acute Liver Injury and Failure. Vet. Clin. North. Am. Small Anim. Pract. 47 (3), 617–630. 10.1016/j.cvsm.2016.11.010 28065578

[B37] Tisma V. S. Basta-Juzbasic A. Jaganjac M. Brcic L. Dobric I. Lipozencic J. (2009). Oxidative Stress and Ferritin Expression in the Skin of Patients with Rosacea. J. Am. Acad. Dermatol. 60 (2), 270–276. 10.1016/j.jaad.2008.10.014 19028405

[B38] Vile G. F. Tyrrell R. M. (1993). Oxidative Stress Resulting from Ultraviolet A Irradiation of Human Skin Fibroblasts Leads to a Heme Oxygenase-dependent Increase in Ferritin. J. Biol. Chem. 268 (20), 14678–14681. 10.1016/s0021-9258(18)82386-9 8325845

[B39] Wang H. An P. Xie E. Wu Q. Fang X. Gao H. (2017). Characterization of Ferroptosis in Murine Models of Hemochromatosis. Hepatology 66 (2), 449–465. 10.1002/hep.29117 28195347 PMC5573904

[B40] Weiss A. Spektor L. Cohen L. A. Magid Gold I. Zhang D. L. Truman-Rosentsvit M. (2018). Orchestrated Regulation of Iron Trafficking Proteins in the Kidney during Iron Overload Facilitates Systemic Iron Retention. PLoS One 13 (10), e0204471. 10.1371/journal.pone.0204471 30321179 PMC6188744

[B41] Wu H. Wu T. Xu X. Wang J. Wang J. (2011). Iron Toxicity in Mice with Collagenase-Induced Intracerebral Hemorrhage. J. Cereb. Blood Flow Metab. 31 (5), 1243–1250. 10.1038/jcbfm.2010.209 21102602 PMC3099628

[B42] Xie Y. Hou W. Song X. Yu Y. Huang J. Sun X. (2016). Ferroptosis: Process and Function. Cell Death Differ 23 (3), 369–379. 10.1038/cdd.2015.158 26794443 PMC5072448

[B43] Yang W. S. Stockwell B. R. (2016). Ferroptosis: Death by Lipid Peroxidation. Trends Cel Biol 26 (3), 165–176. 10.1016/j.tcb.2015.10.014 PMC4764384 26653790

[B44] Yu Y. Jiang L. Wang H. Shen Z. Cheng Q. Zhang P. (2020). Hepatic Transferrin Plays a Role in Systemic Iron Homeostasis and Liver Ferroptosis. Blood 136 (6), 726–739. 10.1182/blood.2019002907 32374849 PMC7414596

[B45] Zargar S. Alonazi M. Rizwana H. Wani T. A. (20192019). Resveratrol Reverses Thioacetamide-Induced Renal Assault with Respect to Oxidative Stress, Renal Function, DNA Damage, and Cytokine Release in Wistar Rats. Oxid Med. Cel Longev 2019, 1702959. 10.1155/2019/1702959 PMC6754927 31583036

[B46] Zhan F. Zhao G. Li X. Yang S. Yang W. Zhou S. (2019). Inositol-requiring Enzyme 1 Alpha Endoribonuclease Specific Inhibitor STF-083010 Protects the Liver from Thioacetamide-Induced Oxidative Stress, Inflammation and Injury by Triggering Hepatocyte Autophagy. Int. Immunopharmacol 73, 261–269. 10.1016/j.intimp.2019.04.051 31121416

